# The impact of uric acid on musculoskeletal diseases: clinical associations and underlying mechanisms

**DOI:** 10.3389/fendo.2025.1515176

**Published:** 2025-02-04

**Authors:** Jing Zhang, Na Sun, Wanhao Zhang, Wenjie Yue, Xiaochen Qu, Zhonghai Li, Gang Xu

**Affiliations:** ^1^ Department of Orthopaedics, The First Affiliated Hospital of Dalian Medical University, Dalian, China; ^2^ Department of Pharmacy, The Third People’s Hospital of Dalian, Dalian, China; ^3^ Key Laboratory of Molecular Mechanism for Repair and Remodeling of Orthopaedic Diseases, Dalian, Liaoning, China

**Keywords:** uric acid, musculoskeletal diseases, sarcopenia, osteoarthritis, intervertebral disc degeneration, gout, osteoporosis

## Abstract

Serum urate (SU) levels are significantly elevated in conditions such as gout, type 2 diabetes (T2D), obesity, and other metabolic syndromes. Recently, due to the high prevalence of hyperuricemia (HUA), numerous clinical connections between SU and musculoskeletal disorders like sarcopenia, osteoarthritis (OA), rheumatoid arthritis (RA), intervertebral disc degeneration (IDD), and osteoporosis (OP) have been identified. This review discusses the mechanisms linking SU to musculoskeletal disorders, as well as the clinical associations of SU with conditions such as sarcopenia, T2D with sarcopenia, McArdle disease, heart failure, gout, OA, IDD, OP and exercise-induced acute kidney injury (EIAKI), offering valuable insights for improved prevention and treatment strategies. Mechanisms linking SU to musculoskeletal disorders include oxidative stress, MSU (monosodium urate) crystal deposition, inflammation, and other factors. In adults, both age and SU levels should be considered for preventing sarcopenia, while gender and SU may directly impact muscle mass in children and adolescents. HUA and gout may be risk factors for OA progression, although some reports suggest otherwise. A U-shaped relationship between SU and IDD has been reported, particularly in Chinese men, indicating lower or higher SU level may be risk factors for IDD. Maintaining SU levels within a certain range may help prevent OP and fractures. Future research, including epidemiological studies and new pathogenesis findings, will further clarify the relationship between musculoskeletal diseases and SU.

## 1 Introduction

Serum urate (SU) levels are known to be significantly elevated in conditions such as gout, type 2 diabetes (T2D), obesity, and other metabolic syndromes (1). It is widely recognized that elevated SU, which is the end product of purine metabolism in humans and other higher primates (2), can pose health risks and lead to adverse outcomes, potentially even death (3). Factors contributing to high SU levels include excessive intake of purine-rich foods, alcohol, and fructose, as well as abnormal purine metabolism and/or reduced renal and intestinal SU excretion (4, 5). When SU concentration exceeds a certain threshold, it is diagnosed as hyperuricemia (HUA) (>7.0 mg/dL in men and >6.0 mg/dL in women). According to a 2015-2016 American epidemiological survey, the prevalence of HUA was 20.2% in men and 20.0% in women, totaling 22.8 million and 24.4 million individuals, respectively (5, 6). In China, the largest developing country, the prevalence of HUA among adults was 8.4% from 2009 to 2010 (7), with a prevalence of 6.4% in middle-aged and older adults according to the CHARLS 2011 survey (8).

Unregulated HUA has been linked to various health conditions such as renal disease (1, 9–13), cardiovascular disease (CVD) (1, 11, 14–16), hypertension (10, 17), T2D (1, 18–20), diabetic kidney disease (21), and others, all of which are associated with increased morbidity and mortality (3). In the musculoskeletal system, on the one hand, persistent presence of supersaturated level of SU can lead to the formation of monosodium urate (MSU) crystals in and around synovial joints, causing severe sterile inflammation/pain known as gout (22). The prevalence of HUA has led to the clinical connections between SU and musculoskeletal disorders like sarcopenia, gout, osteoarthritis (OA), intervertebral disc degeneration (IDD), and more (23–26). OA, for instance, affects a significant portion of the population, with a lifetime risk of 50% and a current prevalence of 10-15% in adults (27). In Chinese older adults aged 65 and above, the prevalence of OA is 8.1% (28), while it is projected to affect 78 million American adults by 2040 (29). Sarcopenia, characterized by muscle mass loss, affects an estimated 9.9%-40.4% of older individuals (30). IDD primarily affects middle-aged and older individuals but is becoming more prevalent at a younger age due to modern lifestyle factors, leading to substantial medical costs and economic burdens globally (31). Exercise-induced acute kidney injury (EIAKI), a hereditary pathological condition frequently developed in patients with renal hypouricemia (RHUC), due to increased renal urate clearance as a result of genetic mutations in the urate transporter URAT1 (RHUC1) or GLUT9 (RHUC2) (32) that reabsorbs urate in the renal proximal tubule. After several hours of exhaustive (anaerobic) exercise, the symptoms of EIAKI include loin pain, nausea and vomiting. It was suggested that after exhaustive exercise, an increase in intraluminal urate in the proximal straight tubule and/or thick ascending limb of Henle’s loop might activate nucleotide-binding oligomerization domain-like receptor family pyrin domain-containing 3 (NLRP3) inflammasome via the luminal Toll-like receptor 4-myeloid differentiation factor 88-phosphoinositide 3-kinase-mammalian target of rapamycin (TLR4-MyD88-PI3K-mTOR) and release interleukin-1β (IL-1β), which might cause the symptoms of EIAKI (32). This review aims to compile recent literature on the involvement of uric acid (UA) in these musculoskeletal conditions and their underlying mechanisms to offer insights for improved prevention and treatment.

## 2 UA: a potential risk factor for musculoskeletal diseases

**Figure 1:**
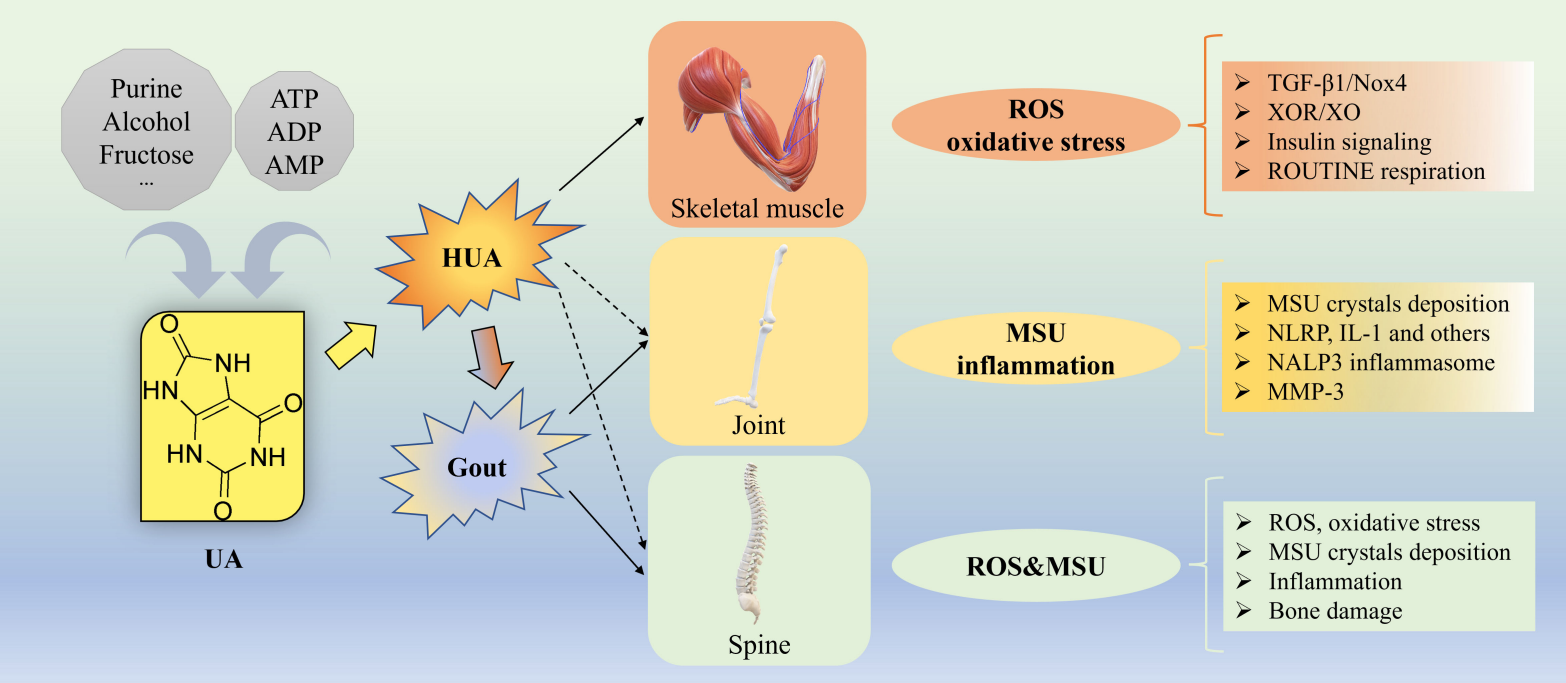
Underlying mechanisms for UA causing musculoskeletal diseases. Approximately 80% of total UA in the human body is endogenously produced from ATP, ADP, and AMP, while the remaining 20% is derived from dietary source such as purine, alcohol, fructose and others. Gout, a common metabolic disorder, is the MSU crystal-induced the most common inflammatory arthritis with episodic flares of intense inflammation related to long standing HUA. For skeletal muscle, the underlying mechanisms of how HUA causes oxidative stress by elevating ROS generation via activation of TGF-β1/Nox4 signaling, XOR/XO, insulin signaling, ROUTINE respiration and others. For joint, the underlying mechanisms of how HUA causes MSU crystal-induced inflammation via NLRP, IL-1, NALP3 inflammasome, MMP-3 and others. For spine, the underlying mechanisms of how HUA elevates the level of ROS and causes oxidative stress, MSU crystals deposition, inflammation, bone damage and others. ADP, adenosine diphosphate; AMP, adenosine 5’-monophosphate; ATP, adenosine triphosphate; HUA, hyperuricemia; IL, interleukin; MMP-3, Matrix Metalloproteinase-3; MSU, monosodium urate; NLRP, nucleotide binding domain and leucin-rich repeat containing protein; Nox4, NADPH oxidase-4; ROS, oxygen species; TGF, transforming growth factor; UA, uric acid, XOR, xanthine oxidoreductase; XO, xanthine oxidase.

Approximately 80% of total UA in the human body is endogenously produced directly from xanthine the metabolic products of adenine nucleotides (ATP, ADP, and AMP), primarily in the liver, while the remaining 20% is derived from dietary purine metabolism. The typical UA pool in the body stores around 1200 mg of UA (33). Daily UA production is approximately 700 mg, with two-thirds excreted by the kidneys, one-third by the intestines, and the remainder by sweat glands. A dynamic equilibrium between UA production and excretion involving urate reabsorption (primarily via urate reabsorption transporters URAT1, GLUT9a/b, OAT10 and OAT4) and secretion transporters (primarily OAT1/OAT3, ABCG2, NPT1/4 and ABCC4) (34, 35) is maintained under normal physiological conditions (5, 36). Exogenous fructose-induced *de novo* increase of UA production in human body (37) as shown in the hepatocyte cell line (HepG2) *in vitro*, and mitochondrial superoxide generation (38), suggest that exogenous fructose-induced UA release from liver cells can lead to oxidative stress in various tissues, including skeletal muscle (39). The underlying mechanisms linking UA to musculoskeletal diseases are summarized in **Figure 1**. Besides, UA can stimulate vascular smooth muscle cell proliferation by entering into cells via a functional urate transporter (40). EIAKI was associated with RHUC due to genetic mutations in the urate transporter URAT1 or GLUT9 (32). In Urat1- urate oxidase (Uox) double knockout mice, the pathogenic mechanism of EIAKI was shown attributed to increased levels of IL-1β via NLRP3 inflammasome signaling and Na^+^-K^+^-ATPase dysfunction involved in excessive urinary urate excretion (41). Additionally, XOR inhibitors appeared to be a potential therapeutic drug for the treatment of EIAKI (41).

### 2.1 Skeletal muscle activity enhances UA and reactive oxygen species production

Skeletal muscle presents a large surface area to plasma urate and plays a dominant role for the development of insulin resistance (IR)/T2D. In skeletal muscle, fructose-induced increase in purine nucleotide degradation and purine release from differentiated muscle cells (42), is a major source of purine metabolites (such as hypoxanthine and xanthine) that can lead to the increased production of UA in liver cells and endothelial cells that express xanthine oxidoreductase (XOR) (42). The skeletal muscle of male Sprague-Dawley (SD) rats was shown to have allopurinol-sensitive xanthine oxidase (XO) activity and synthesize UA (43). Exposure of rat skeletal muscle cells/myotubes (L6) *in vitro* to high fructose was shown to stimulate mitochondrial ROS and nitric oxide (NO) production, and the expression of inducible NO synthase (44). HUA-induced upregulation of transforming growth factor-β1 (TGF-β1), was shown to induce the NADPH oxidase isoform, NOX4 expression in freshly isolated human pulmonary artery smooth muscle cells (HPASMC) (45, 46). In HepG2 cells, UA produced by XO activity was shown to induce mitochondrial superoxide generation by translocating the NOX4, to the mitochondria (38). While UA is known to possess antioxidant properties, elevated UA production can paradoxically exert oxidative damage through generation of ROS (47, 48). HUA is strongly associated with IR, obesity and metabolic syndrome. Lowering UA levels in mice using allopurinol has been shown to improve insulin sensitivity in obese Pound mice with metabolic syndrome by improving proinflammatory endocrine imbalance in the adipose tissue (49). In potassium oxonate-induced acute HUA mouse model, very poor response to insulin activation of Akt in muscle, liver, and fat tissues, is indicative of HUA induced oxidative stress and IR (50). Exposure of differentiated mouse myoblasts (differentiated from mouse myoblast cell line, C2C12) to UA at a concentration of 15 mg/dL resulted in increased ROS production, inhibition of IRS1-AKT signaling, suppressed insulin-stimulated glucose uptake, and induction of IR (51). Similarly, exposure of differentiated C2C12 myotubes to UA at 12.6 mg/dL led to a 32% increase in oxidative stress, a 135% increase in antioxidant capacity, and a nearly 237% rise in triglyceride content. Additionally, UA reduced endogenous ROUTINE respiration, complex II-linked oxidative phosphorylation (OXPHOS), and electron transfer system (ETS) capacities in these myotubes, indicating detrimental effects of excessive UA on skeletal muscle (52) (**Figure 1**).

### 2.2 HUA affects joints and soft tissues

The proposed four distinct stages of pathological basis of gout involves four distinct stages: asymptomatic HUA, acute attacks, intercritical phase, and chronic tophaceous gout (53). Initially, asymptomatic HUA and acute attacks occur interchangeably, leading to polyarticular attacks in the chronic phase where symptoms include alternating attacks and MSU crystal deposition (tophi, typically white sand-like crystals) within joints typically the first metatarsophalangeal joint, ankle, knee, and elbow, periarticular and extra-articular sites of soft tissues (e.g., tendons, ligaments, muscles, cartilage) that are located outside of joints, ultimately resulting in OA and joint damage (54). MSU crystallizes when its plasma concentration exceeds its solubility (around 7 mg/dl, or 420 μmol/l). The acute gouty attack is called an acute sterile inflammation. MSU is widely accepted as a classical activator of the nucleotide-binding domain and leucine-rich repeat-containing protein 3 (NLRP3) linked to the development of gout (55, 56). Phagocytosis of MSU crystals particularly by neutrophils and macrophages, leads to the generation of ROS through activation of NADPH oxidases (52). This event activates the NLRP3 inflammasome which is a cytoplasmic multiprotein complex comprising a sensor protein, an adaptor protein ASC (apoptosis-associated speck-like protein containing a caspase-recruitment domain), and pro-caspase-1. When the inflammasome complex is assembled through a homotypic interaction between PYD and CARD domains of sensor proteins and ASC (apoptosis-associated speck-like protein containing a caspase-recruitment domain), inactive pro-caspase-1 is auto-processed to active caspase-1, leading to maturation of pro-interleukin-1β (pro-IL-1β) or pro-IL-18 to their active forms and released into the extracellular environment (55). Colchicine, that has been the drug of choice for gout attack for more than 200 years, that blocks MSU crystal-induced IL-1β generation upstream of NLRP3 inflammasome activation possibly by suppressing the microtubule-mediated NLRP3 inflammasome assembly (55, 56).

Several studies have investigated the signaling pathways in macrophages and neutrophils activated by MSU crystals, using animal experiments to identify potential therapy targets in the MSU-induced signaling pathway. IL-1β, a key inflammatory factor in gout, has been targeted for therapy and can be secreted by various immune cells such as macrophages, neutrophils, and mast cells. The processing of pro-IL-1β by caspase-1 in the NLRP3 inflammasome leads to the formation of mature IL-1β (57). SLC3037, an NLRP3 inhibitor that blocks NEK7 [never in mitosis A (NIMA)-related kinase 7 (NEK7) critical in NLRP3 oligomerization] binding to NLRP3, shows promise as a novel agent for diseases associated with NLRP3 inflammasome activation like gout (58). Experimental systems involving NLRP3 inflammasome activation in mouse bone marrow macrophages, along with *in vivo* mouse models, have been utilized to study the effects of Euodiae fructus (ER), a traditional Chinese medicine, on UA and gout. Results suggest that ER is a potent NLRP3 inhibitor, exerting anti-HUA and anti-gout effects by reducing UA production, enhancing UA excretion, and alleviating inflammation, partly through NLRP3 inflammasome inhibition (59). Furthermore, the role of T cells in gout is gaining recognition, with evidence indicating their involvement in both adaptive and innate immune responses. While T cells are not directly implicated in the inflammation caused by MSU crystals, they may have proinflammatory or anti-inflammatory regulatory functions in gouty inflammation (57).

Matrix Metalloproteinase-3 (MMP-3) has been studied in relation to the development of gouty arthritis from HUA. Research has shown that high levels of UA can lead to an increase in MMP-3, which in turn promotes the degradation of proteoglycan and induces the crystallization of MSU crystals (60). Genetic variants in UA transporters (such as GLUT9, URAT1, ABCG2, OAT1 and OAT3) have been linked to HUA (34, 61–65), increasing the risk of MSU crystal precipitation. The inflammatory response triggered by these crystals can cause cartilage damage, potentially leading to OA. A case-control study involving 243 unrelated Mexican-mestizo individuals revealed significant associations for 8 specific single nucleotide polymorphisms (SNPs) related to various genes. Gene-gene interactions were also observed, highlighting synergistic or antagonistic effects on OA development. It was found that common gene variants associated with UA transport were linked to OA in the Mexican population (66). Since MSU crystals are more likely to form at lower temperatures, recently by leveraging mild hyperthermia to synergistically enhancing catalytic decomposition of UA and endogenous hydrogen peroxide (H_2_O_2_) with polydopamine-platinum nanoparticle was shown to be a potential therapeutic strategy for the management of acute gout (67) (**Figure 1**).

### 2.3 HUA: a risk factor for spinal inflammation

The incidence of spinal gout (with symptoms of fever, back pain and spine compression that occur within a short time frame and weakness) is more common than previously thought, in patients with extensive prior history of gout or HUA. In spinal gout, the lumbar spine is the more commonly affected (68) than cervical vertebrae or thoracic vertebrae. Spinal gout can be identified by magnetic resonance imaging (MRI with gadolinium contrast), computed tomography (CT) and positron emission tomography (PET) (68). Patients with HUA or gout can have the presence of tophi in the intervertebral disc (IVD) or endplate, and these patients also exhibited severe IDD (4). Therefore, it was speculated that MSU crystals accumulation in the endplate and IVD may lead to mechanical damages disrupting the supply of nutrients and oxygen to the IVD and trigger inflammatory reactions through oxidative stress in mitochondria exacerbating IDD (69). The extracellular matrix (ECM) of the IVD contains a variety of proteoglycans that possess multiple negative charges. The anion gap caused by negatively charged proteoglycans can significantly increase the solubility of MSU (69) and thus prevent its crystallization. Therefore, it was also speculated that destruction of the proteoglycans by MSU-induced MMPs can further decrease the solubility of MSU and help grow tophi into larger structures (69) (**Figure 1**).

### 2.4 HUA: an important player in osteoporosis

OP is the most common metabolic musculoskeletal disorders primarily in postmenopausal women with estrogen deficiency, diabetic patients, and obese patients, affecting about 200 million people worldwide. OP is characterized by reduced bone mineral density (BMD), reduced bone mass and destruction of bone microarchitecture, leading to increased bone fragility and increased risk of fracture. The clinical manifestation of OP includes joint inflammation/pain, stiffness, and reduced mobility. Common risk factors of OP are aging, sex hormone deficiency, calcium and vitamin D deficiency, elevated parathyroid hormone (PTH) levels, genetics, and unhealthy lifestyle (70). Moreover, oxidative stress caused by excessive accumulation ROS produced in mitochondria also plays a pivotal role in the pathogenesis of OP (70). Excessive accumulation of ROS can damage cell membranes, cytoplasmic structures, and even DNA. Excessive ROS can inhibit the differentiation and proliferation of osteoblasts (OB) (70). Multiple studies also show that disorders in purine metabolism may cause various type of osteoporosis. Abnormal purine metabolism causes HUA and the accumulation of ROS. HUA and gout also increase the risk of bone fragility/fracture because MSU-induced oxidative stress and proinflammatory cytokines (e.g., IL-1β, TNFα, IL-6, IL-8) released by MSU-phagocytosed monocytes, increase bone resorption and decrease bone formation (71). For bone remodeling, osteoclasts (OC), OB, and osteocytes play important roles. OB regulate OC differentiation and activation through receptor activator of nuclear factor kappa-β ligand (RANKL)-mediated signaling pathways. The RANKL receptor (called RANK) is expressed in OC progenitors to promote OC differentiation and maturation. 1,25 vitamin D, parathyroid hormone (PTH), and IL-6 activate RANKL-signaling in OB. Both proinflammatory cytokines and oxidative stress, induced by MSU, stimulate OC activity and inhibit OB function, which result in bone loss (71). MSU was shown to directly inhibit 1α-hydroxylase expression in renal proximal tubules and thereby reduce the active form of vitamin D, 1,25-dihydroxyvitamin D3 1,25(OH) concentrations in hyperuricemic SD rats (72). In postmenopausal women, HUA is associated with vitamin D insufficiency (73). The impact of SU on bone health also depends on the hormonal status in postmenopausal women (74). The SU level was shown positively correlated with elevated parathyroid hormone (PTH) levels in SD rats (71, 72). Therefore, UA has the ability to inhibit 1,25-dihydroxyvitamin D3 1,25(OH) production, leading to secondary hyperparathyroidism and further worsening UA-related bone loss (70). Additionally, using simple hyperuricemic group and HUA castration group of male Wistar rats, it has been shown that the castration group of hyperuricemic rats had significantly decreased level of SU and serum phosphorus suggesting androgen might have positive impact on UA levels and bone metabolism/growth in presence high SU level (75). In US adult females aged 40-60 years, testosterone level was shown positively associated with lumbar BMD (76).

### 2.5 HUA: a potential risk factor in rheumatoid arthritis

In the context of RA, a polyarticular systemic autoimmune disease characterized by the presence of autoantibodies, the role of UA remains unclear. RA is a musculoskeletal disease characterized by progressive joint cartilage and bone destruction, inflammation affecting muscle, bone, lungs, exocrine glands, liver and brain, chronic synovial inflammation and hyperplasia, production of autoantibodies including rheumatoid factor (RF) and anti-citrullinated protein antibody (ACPA), physical impairment, work disability and early morbidity and mortality (77). RA is different from OA because RA is an autoimmune disease in which the body’s own immune system attacks the body’s joints and OA is caused by mechanical wear and tear on joints. Sarcopenia, a progressive and generalized skeletal muscle disorder involving accelerated loss of muscle strength and mass, affects approximately one in four people with RA (78). Hypertension is highly prevalent among RA patients. Rats treated with 2% oxonic acid was shown to develop mild HUA and hypertension indicating increased SU levels can promote the development of hypertension and systolic blood pressure (79) most likely by causing vascular smooth muscle cell proliferation as shown in an *in vitro* experiment (79). The HUA and hypertension were partially inhibited by losartan (an angiotensin II receptor blocker) suggesting involvement of activation of the renin-angiotensin system in developing hypertension. Allopurinol (a xanthine oxidase inhibitors) treatment also prevented the hyperuricemia and hypertension (79) suggesting role of HUA in developing hypertension. Allopurinol treatment in adults with hypertension and HUA also effectively lowered SU levels and hypertension (80). The prevalence of metabolic syndrome, caused by hyper-insulinemia/IR that stimulates UA reabsorption in the proximal tubule (81), is as high as 40% in RA patients (82) suggesting potential role of HUA in developing RA. HUA causes hypertension and CVD, and CVD is associated with renal dysfunction in patients with RA (83) again indicating potential role of HUA in developing RA. Endothelial dysfunction is also common in RA (84) and UA was shown to promote endothelial dysfunction and atherogenesis (85). Thus, it appears that UA is not just an innocent bystander, it could be an active player in CVD-associated mortality in patients with RA. UA was shown as a strong correlate of renal dysfunction in RA patients (83). Deposition of MSU crystals can promote cartilage degradation in the joints (especially metatarsophalangeal joint, mid-foot, knee and finger distal interphalangeal joints) (86, 87). Levels of serum proinflammatory cytokines IL-1β, IL-6, IL-17 and TNFα were shown significantly higher in patients with RA (88, 89). Peripheral blood mononuclear cells, pre-treated with soluble UA and stimulated by MSU crystals, were also shown to enhance IL-1β, IL-6 production (90).

## 3 Underlying clinical correlations for SU and musculoskeletal diseases

### 3.1 SU and skeletal muscle diseases

Abnormal concentration of SU is related to some skeletal muscle and metabolic diseases, such as sarcopenia, T2D, McArdle disease (MCD), heart failure and peritoneal dialysis and others (91–94).

#### 3.1.1 SU levels and sarcopenia

Sarcopenia is defined as a gradual reduction in skeletal muscle mass and function (95). Elevated levels of SU may act as a protective mechanism against free radical damage, potentially preventing muscle protein damage and loss of muscle mass and strength in the elderly population (96). A community-based cross-sectional study using 3079 Chinese adults (963 men and 2116 women) aged 40-75 years revealed that higher SU levels were linked to increased BMD and muscle mass, with the relationship between SU and BMD being influenced by muscle mass (97). Additionally, research on individuals aged 92.8 ± 3.1 years indicated that higher SU levels were associated with improved muscle function, suggesting a potential role of UA in slowing the progression of sarcopenia (98).

A recent cross-sectional study conducted in western China with 4236 adults aged 50 years and older found a negative correlation between SU levels and sarcopenia in both men and women (23). Additionally, the study revealed an inverted J-shaped correlation between SU levels and handgrip strength in women, as well as a positive correlation between SU quartiles and skeletal muscle index regardless of gender. The study recommended that Chinese adults aged 50 years and older with higher SU levels (men: <5.36, 5.36–6.25, 6.25-7.12, ≥7.12 mg/dL; women: <4.26, 4.26–4.94, 4.94-5.72, ≥5.72 mg/dL) may experience increased muscle mass and grip strength, potentially slowing down the progression of sarcopenia (23), which is consistent with previous research findings (97, 98). Furthermore, gender differences in SU levels during adolescence in children and adolescents were noted, likely influenced by sex hormones and pubertal development, with muscle mass playing a role in this variation (99). In this cross-sectional study involving 823 children and adolescents aged 6-18 years of both sexes found that muscle mass contributed to 7.7% and 43.0% of SU variability in girls and boys, respectively (99) (**Table 1**).

**Table 1:** Cross-sectional studies on correlation between UA and skeletal muscle (sarcopenia).

Year	Subjects	Age (years)	UA (mg/dL)	Correlation	Ref.
2008	497 Italian adults (226 men and 271 women)	76.0 ± 5.4	<4.4, 4.4–5.5, **>5.5**	higher levels of SU, a protective response	(96)
2009	7544 American men and women	≥40	<6, 6-7, 7-8, **>8**	abnormally high SU levels, sarcopenia	(91)
2013	630 Japanese men	≥30	<5.4, 5.4-6.0, 6.1-6.8, **>6.8**	HUA, poor muscle strength, an overturned J-shaped correlation	(100)
2016	3079 Chinese adults (963 men and 2116 women)	40-75	4.32, 5.34, 6.15, **7.57**	higher SU levels, increased muscle mass	(97)
2017	239 Italian adults (73 men and 166 women)	92.8 ± 3.1	<4.8, 4.8–6.1, >**6.1**	higher SU levels, better muscle function	(98)
2018	3595 American adults (1857 men and 1738 women)	≥20	men, <5.5, 5.6-6.5, ≥6.6; women, <4.2, 4.3-5.2, ≥5.3	aged 20 to 40, SU, muscle strength, a negative correlation; above 60 years, reversed	(101)
2020	823 Brazilian children and adolescents (463 boys and 360 girls)	6-18	boys, 3.5 ± 0.8, 4.3 ± 1.1, 5.3 ± 1.0; girls, 3.5 ± 0.9, 3.9 ± 0.9; 4.0 ± 1.0	gender differences in SU occur during adolescence, a direct effect of muscle mass	(99)
2022	4260 Chinese adults (1542 men and 2718 women)	≥50	men, <5.36, 5.36–6.25, 6.25-7.12, **≥7.12**; women, <4.26, 4.26–4.94, 4.94-5.72, **≥5.72**	higher SU levels, increased muscle mass and grip strength	(23)

HUA, hyperuricemia; SU, serum urate; UA, uric acid.

Bold it is diagnosed as HUA (>7.0 mg/dL in men and >6.0 mg/dL in women).

**Figure 2:**
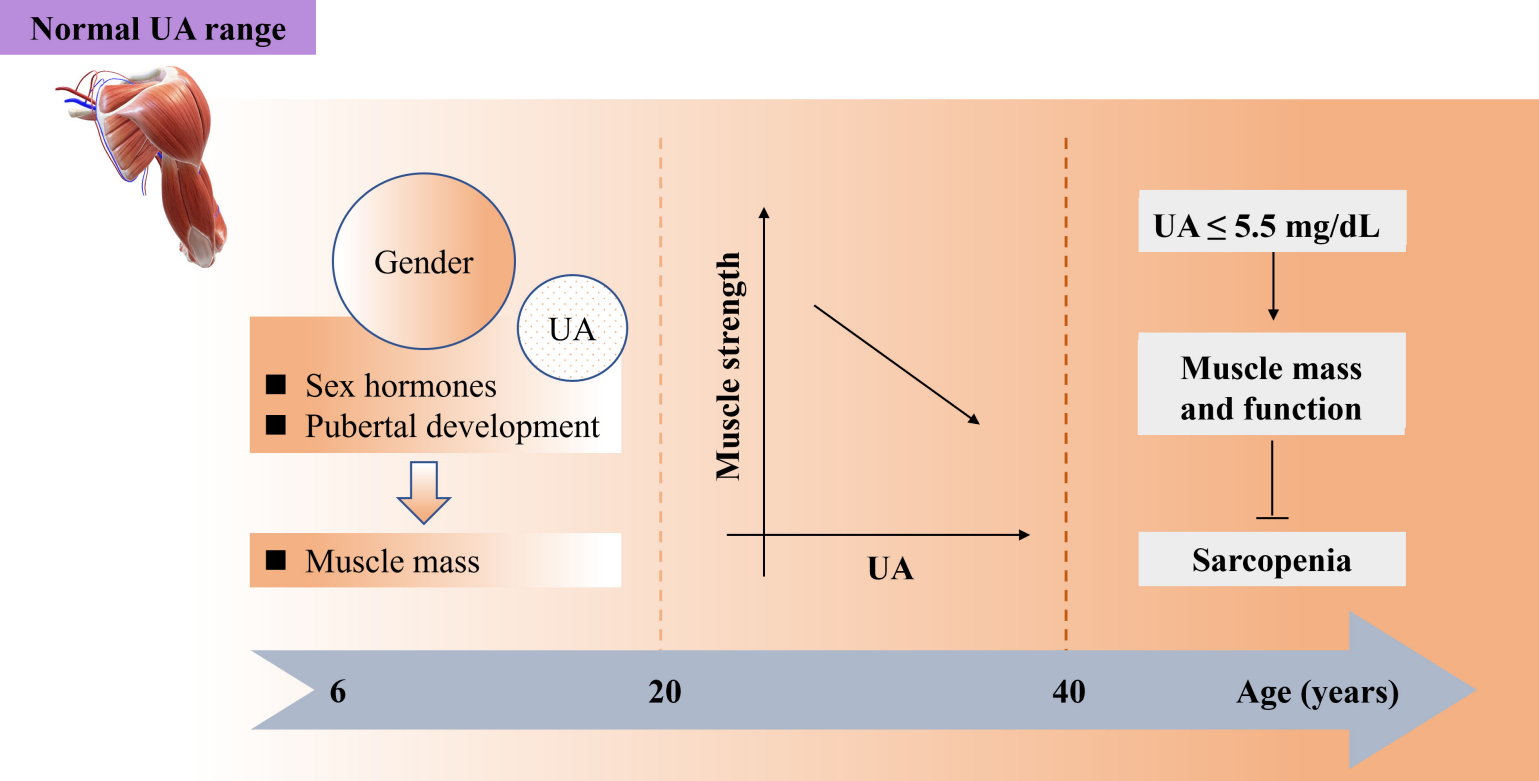
Both age and SU play important role for maintaining normal function of skeletal muscles preventing sarcopenia while SU stays within normal range. SU, serum urate.

However, abnormally high SU levels (>8 mg/dL) are linked to increased production of ROS and inflammation, both of which contribute to sarcopenia (91). SU at higher concentrations exhibits oxidant properties that are associated with systemic inflammation, leading to a higher risk of muscle strength loss. A cross-sectional study involving 630 Japanese men (aged ≥ 30 years) examined the relationship between SU concentration, grip strength, and leg extension power. The results indicated that HUA (>7.0 mg/dL) was related to poor muscle strength, demonstrating an inverted J-shaped correlation between SU quartiles and muscle strength (100). As age plays a significant role in skeletal muscle composition and function, the adverse effects of UA are more pronounced in younger individuals compared to older individuals. The association between SU levels (men: <5.5, 5.6-6.5, ≥6.6 mg/dL; women: <4.2, 4.3-5.2, ≥5.3 mg/dL) and muscle strength varied across age groups. While a negative correlation was observed in adults aged 20 to 40 years, this relationship reversed in individuals above 60 years old. It is essential to consider both age and muscle strength when setting SU goals for individuals with HUA (101). Both age and SU levels should be considered when evaluating skeletal muscle function and preventing sarcopenia, aligning with the notion that sarcopenia involves age-related muscle mass loss influenced by various factors. For middle-aged and older individuals (≥40 years), higher SU levels (≥5.5 mg/dL) may help safeguard muscle mass and function against sarcopenia. Conversely, younger individuals (aged 20 to 40 years) exhibit a negative correlation between SU levels and muscle strength. In children and adolescents (aged 6 to 18 years), gender and SU levels could directly impact muscle mass. Moreover, excessively high SU levels (>7.0 mg/dL) could contribute to sarcopenia by promoting increased ROS and inflammation (**Table 1**, **Figure 2**).

#### 3.1.2 HUA: a risk factor for T2D and sarcopenia

UA is primarily produced in the liver and to a lesser extent in small intestine (102), adipose tissue (103), and endothelial cells (42), and is predominantly excreted through the urinary tract and sightly through intestine and sweat glands. Several factors can contribute to HUA, including a diet rich in fructose (104), the consumption of exotic organic products (47), and alcohol intake (105, 106). HUA is often associated with metabolic syndrome, which is characterized by abdominal obesity, glucose intolerance, IR, dyslipidemia, and hypertension (47). Elevated SU levels play a role in regulating oxidative stress, inflammation, and enzymes involved in glucose and lipid metabolism, contributing to the deterioration of metabolic homeostasis (47). In fact, HUA and abdominal obesity was shown to synergistically increase the risk of hypertriglyceridemia and low HDL-C in both sexes (107). It is still debatable whether high SU levels are a strong independent predictor of diabetes and precede the development of IR and diabetes (108), because results from some studies indicate IR/hyperinsulinemia might precede HUA (81, 109, 110). Obese individuals with elevated SU levels tend to have lower insulin sensitivity and higher levels of oxidative stress compared to those with normal SU levels (111). A cohort study involving 440 non-diabetic Caucasian subjects with higher SU levels (6.4 ± 0.9) showed a weaker metabolic phenotype and lipid profile, increased 2-hour post-load glucose levels, elevated fasting and 2-hour post-load insulin levels, reduced skeletal muscle insulin sensitivity, and impaired hepatic insulin sensitivity (112) (**Table 2**).

**Table 2:** Cross-sectional and cohort studies on correlation between UA and skeletal muscle (others).

Year	Subjects	Disease	UA (mg/dL)	Correlation	Ref.
2017	a Canadian cohort study, Twelve subjects with MCD and Twelve control subjects (8 men and 4 women, separately)	MCD	HUA	blocked glycogenolysis, myogenic HUA and increased oxidative stress via the xanthine oxidase pathway	(92)
2018	a Caucasian cohort study, 440 non-diabetic subjects (199 men and 241 women)	T2D	3.5 ± 0.5, 4.7 ± 0.3, 6.4 ± 0.9	higher SU levels, reduced skeletal muscle insulin sensitivity and impaired hepatic insulin sensitivity	(112)
2021	a Japanese cross-sectional study, 401 subjects with T2D (209 men and 192 postmenopausal women)	T2D and sarcopenia	men, 5.5 ± 1.4; women, 4.6 ± 1.3	higher SU, an independent risk factor of reduced muscle mass in men with T2D	(93)
2021	a Chinese cross-sectional study, 3393 subjects (1469 men and 1924 women)	obesity	men, 5.64 ± 0.85, 7.87 ± 0.79; women, 4.37 ± 0.77,6.64 ± 0.64	HUA in women, the visceral fat area to leg muscle mass ratio positively related	(113)
2020	a Chinese retrospective cohort study, 802 subjects (464 men and 338 women)	peritoneal dialysis	5.5 ± 0.7, 6.8 ± 0.3, 8.2 ± 0.9	SU levels in middle-aged and elderly peritoneal dialysis male patients, positively correlated with appendicular skeletal muscle mass	(119)
2020	a Brazilian cross-sectional study, 113 subjects (75 men and 38 women)	kidney transplant	3.1–7.0, 6.1–13.4	SU levels, positively associated with muscle mass, not with functional capacity	(120)
2022	an Australian cohort study, 409 older subjects (205 men and 204 women)	OA	5.74 ± 1.38	over 10 years, the increased SU, involved in the decline in leg strength	(121)

HUA, hyperuricemia; MCD, McArdle disease; SU, serum urate; T2D, type 2 diabetes; UA, uric acid.

Skeletal muscle plays a critical role in the effects of insulin and the development of T2D, making sarcopenia a common complication in individuals with diabetes. HUA is frequently seen in conjunction with T2D. A cross-sectional study involving 401 subjects with T2D (209 men and 192 postmenopausal women) was conducted to explore the relationship between SU levels and muscle mass. The study found that higher SU levels were an independent risk factor for men with T2D and sarcopenia (93). Furthermore, a strong positive association was observed between obesity, visceral fat accumulation, and HUA. Another cross-sectional study involving 3393 Chinese participants (43.3% men) investigated the ratio of visceral fat area to leg muscle mass and SU levels. The results indicated a positive correlation between the visceral fat area to leg muscle mass ratio and the risk of HUA in women, even after adjusting for confounding variables. Notably, pre-menopausal women exhibited a higher risk of HUA compared to postmenopausal women as the visceral fat area to leg muscle mass ratio increased. The study also revealed that the highest risk of HUA was observed in individuals with both high visceral fat area and leg muscle mass, placing them in the highest quartile of risk (113) (**Table 2**).

#### 3.1.3 HUA and McArdle disease

MCD, also called Type V glycogen storage disease, is an autosomal recessive hereditary condition that results from the lack of glycogen phosphorylase enzyme (the enzyme required for efficient glycogen breakdown) activity in skeletal muscle. Patients with MCD are exercise intolerant because of muscle weakness, fatigue and cramp/pain during moderate- to high-intensity exercise (114). MCD patients often develop HUA because muscular exercise accelerates the degradation of muscle purine nucleotides into inosine and hypoxanthine in the blood that serve as substrates for the synthesis of uric acid in cells of tissues/organ (e.g., liver cells and endothelial cells) expressing xanthine oxidase enzyme, leading to HUA (115). Increased synthesis of UA produces H_2_O_2_ causing increased oxidative stress. Patients with MCD have elevated level of markers of oxidative stress (8-isoprostanes and protein carbonyls) with insufficient levels of compensatory antioxidant enzyme (mitochondrial superoxide dismutase) activity to fully protect skeletal muscle from oxidative damage. Oxidative stress in McArdle patients can potentially lead to rhabdomyolysis and the subsequent development of fixed myopathy (92) (**Table 2**).

#### 3.1.4 XO: a potential link between skeletal muscle abnormality and heart failure

Patients with HF are intolerant to exercise partly due to skeletal muscle abnormalities caused by ROS. In ischemic tissue, higher activity of XO, that catalyzes UA production from xanthine and hypoxanthine, overproduces ROS (H_2_O_2_) (36). Skeletal muscle atrophy is a condition of loss of muscle mass caused by various diseases such as diabetes, heart failure, and sarcopenia, resulting in the reduction of total adenine nucleotides, including ATP, ADP, and AMP involved in cellular signaling (36). The elevated level of XO and degradation products such as hypoxanthine, xanthine, in the atrophic skeletal muscle leads to the over production of UA in XO-expressing tissues (36, 116). Higher SU level in patients with heart failure is considered as an independent marker of prognosis and poor exercise tolerance, partly attributed to abnormal skeletal muscle function. In mouse models of HF, myocardial infarction (MI) induced by ligating the left coronary artery, led to skeletal muscle abnormalities, exercise intolerance and eventually to HF (94) and inhibition of XO by febuxostat/allopurinol was shown to prevent XO-derived ROS (H_2_O_2_) overproduction in XO-expressing tissues and skeletal muscle abnormalities and exercise intolerance (94, 117) suggesting inhibition of XO-derived ROS production can prevent skeletal muscle abnormalities.

#### 3.1.5 Association of SU levels with skeletal muscle strength

In hemodialysis patients, SU level was suggested as a good marker positively correlated with body composition parameters, muscle function, inflammation, and quality of life body composition (118). In a single-center retrospective cohort study on 802 peritoneal dialysis patients (with 464 men and an average age of 46.2 ± 14.2 years), the SU levels in middle-aged and elderly male patients were shown positively associated with appendicular skeletal muscle mass, suggesting a significant impact of appendicular skeletal muscle mass on the relationship between SU and peritoneal dialysis-related mortality (119) (**Table 2**). Additionally, in a cross-sectional study on 113 Brazilian kidney transplant patients (75 men and 38 women), SU level was shown positively correlated with muscle mass (fat-free mass, appendicular muscle mass, muscle mass index, and appendicular muscle mass index) and strength, but not to functional capacity (120) (**Table 2**). A study conducted on a Tasmanian cohort of older adults, consisting of 409 individuals aged 50-79 years (205 men) over the 10-year period, showed association of elevated level of asymmetric dimethylarginine (ADMA) in the blood with the decrease in hand grip and knee extension strength (121). Furthermore, increase in SU level was shown associated with the decline in leg muscle strength over the same 10-year period (121). In a genome-wide association study (GWAS), a single nucleotide polymorphism (SNP) rs1125718 near the WISP1 gene was shown associated with ADMA levels (121). These findings suggest that higher levels of ADMA and SU at baseline may contribute to age-related muscle strength decline, highlighting potential new targets for preventing muscle strength loss over time (121) (**Table 2**).

#### 3.1.6 RHUC involved in EIAKI

Renal hypouricemia (RHUC) was shown as an important risk factor for EIAKI and urinary stones (32). There are two types of RHUC described, RHUC1 and RHUC2. RHUC1 is caused by mutations in the *SLC22A12* gene (encoding the urate reabsorption transporter URAT1), while RHUC2 is caused by mutations in the *SLC2A9* gene (encoding the urate reabsorption transporter GLUT9 isoforms, GLUT9a and GLUT9b). A single−center study from China reported that a proportion of 10 out of 19 patients suffered from EIAKI, the prognosis of which was favorable (122). A large-scale cross-sectional study from Japan found that genetic hypouricemia in men is more strongly involved in EIAKI than transient hypouricemia which is mostly influenced by female hormones (123). Another large-scale cross-sectional study of Japanese schoolchildren discovered that hypouricemia in children including RHUC, as a main cause of EIAKI, was a crucial clinical problem, over and above HUC. The school-age children were investigated, who were younger than the overwhelming majority of people with EIAKI, for reducing the risk of subsequent EIAKI via offering more care and guidance (124). It was suggested that after exhaustive exercise, RHUC patients develop higher concentration of urate in the lumen of proximal straight tubule and/or thick ascending limb of Henle’s loop that could stimulate the luminal TLR4-MyD88-PI3K-mTOR to activate the NLRP3 inflammasome and release IL-1β, which might cause the symptoms of EIAKI (32).

### 3.2 UA, gout and OA

#### 3.2.1 UA: a potential risk factor for OA

Osteoarthritis (OA), one of the top two leading causes of disability worldwide affecting about 7% of the global population, is a chronic, heterogeneous and degenerative joint disease characterized by degeneration of articular cartilage, sclerosis of subchondral bone (just below the cartilage), and osteophyte (growth of bone at the edges of joints/spine) formation, which is considered a leading cause of activity limitation and disability among the elderly population. OA has multiple clinical phenotypes such as abnormal joint tissue metabolism followed by cartilage degradation, bone remodeling, osteophyte formation, joint inflammation and loss of normal joint function (125). The diverse overlapping components of molecular endotypes of OA include bone and cartilage, inflammatory, metabolic and low repair (126). The etiology of OA is multifaceted, involving a complex interplay of metabolic, genetic, mechanical, and inflammatory factors (127). The inflammation of OA could be independent of NLRP3 activation because stress-induced cartilage degradation, in OA cartilage explants from OA patients, was reported not linked to increased IL-1β production (128). NLRP3-deficient mice also showed similar cartilage degradation upon stimulation by inflammatory agents like LPS, IL-1α, and tumor necrosis factor (TNF)α-induced activity of MMP-3, MMP-9, and MMP-13 in cultured chondrocytes and in NLRP3(-/-) chondrocytes (128). Positive associations between SU levels and OA were reported by several studies (129–131) especially in patients of hip or knee OA with elevated SU levels (132, 133). Additionally, it was reported that SU levels may serve as a biomarker for OA progression (134) as SU levels can differ between patients with non-progressive and progressive OA over time, as demonstrated by changes in X-ray joint space narrowing (134). Recently, in a prospective cohort study with a total of 4671 American participants (age range: 20 to 85 years old), including 2988 females and 1683 males, a nonlinear J-shaped relationship between SU levels (with the cut-off values of 5.6 mg/dl for females and 6.2 mg/dl for males) and all-cause mortality in females and males with OA has been reported (135). However, recent data from the Seventh Korea National Health and Nutrition Examination Survey in 2016 suggested no significant association of SU levels with OA in the Korean population, as some patients with OA actually had lower SU levels compared to those without OA (136) (**Table 3**).

**Table 3:** Update of clinical studies on correlation between UA or gout and OA or other bone diseases.

Year	Subjects	Disease	UA (mg/dL)	Correlation	Ref.
2007	a British cross-sectional study, 164 subjects (5904 joint sites)	OA	gout	gout attacking sites, involved in OA	(140)
2008	a British case-control study (164 cases, 656 controls)	OA	gout	gout, involved in knee pain	(141)
2015	an American cross-sectional study, 75 subjects (25 UA, 25 gout and 25 controls)	OA	gout	gout, involved in OA; OA in gout patients, more serious	(143)
2016	a Chinese cross-sectional study, 4685 subjects (2451 men and 2234 women)	OA	men, 6.08 ± 1.38;women, 4.63 ± 1.12;	SU, involved in OA	(133)
2016	a British cross-sectional study, 274 subjects (53 cases, 221 controls)	OA	gout	gout, not involved in OA (radiographic hand, knee or foot).	(142)
2016	a British case-control study (39111 cases, 39111 controls)	OA	gout	gout, involved in a 1-, 2-, 5-, and 10-year risk of OA	(144)
2017	an American cohort study, 88 subjects (29 men and 59 women)	OA	6.30 ± 0.22	SU, involved in joint space narrowing and OA	(134)
2017	a Singapore cohort study, 51858 subjects (2090 gout)	OA	gout	gout, involved in total knee replacement for OA in women but not in men	(24)
2017	a Chinese cohort study, 32723 subjects (27000 men) with gout and 65446 control subjects (54000 men)	rotator cuff tears	gout	Patients with gout, (age ≤50 years) without hypouricemic medication control, a relatively higher risk of receiving rotator cuff repair surgery	(153)
2018	a Korean cross-sectional study, 5842 subjects (2500 men and 3342 women)	OA	5.1 ± 0.02	SU, not involved in OA	(136)
2019	an American cross-sectional study, 2213 subjects (asymptomatic HUA, n=412)	OA	>6.8, without gout	asymptomatic HUA in adults (aged >60 years) without obesity, involved in OA risk	(27)
2021	a Korean longitudinal cohort study (for radiography, n=296; for MRI, n=223)	OA	≥6.0, ≥6.8	SU, not a risk factor for knee OA progression	(152)
2021	a Chinese retrospective case-control study, 1375 subjects (LDH, n=691)	IDD	LDH, 5.70 ± 1.7; non-LDH, 5.68 ± 1.5	both lower and higher SU levels, the LDH rate higher, a U-shaped relationship between SU and IDD, especially among men	(26)
2024	A two-sample MR analysis using summary-level data from GWAS of SU concentration (n= 13585994 European ancestry) and IDD (n=16380337 European ancestry).	IDD	SNPs significantly associated with SU concentration (*p* < 5×10^-8^)	no causal relationship between SU concentration and IDD	(158)

GWAS, genome-wide association studies; HUA, hyperuricemia; IDD, intervertebral disc degeneration; LDH, lumbar disc herniation; MR, Mendelian randomization; MRI, magnatic resonance imaging; OA, osteoarthritis; SU, serum urate; UA, uric acid.

#### 3.2.2 The relationship between gout and OA

OA affects about 30 million individuals and gout affects about 8.3 million individuals though about 43 million individuals in the U.S. are diagnosed with HUA (27). OA is a low-grade inflammatory condition primarily caused by wear and tear on joints that causes cartilage erosion symmetrically on both sides of the body leading to morning stiffness for short period of time with little or no swelling. Gout, a common metabolic disorder, is the MSU crystal-induced the most common inflammatory arthritis with episodic flares of intense inflammation related to long standing HUA (137). Tophaceous gout occurs when MSU crystals form white deposits called tophi around joints and soft tissues causing restricted joint function. Deposition of MSU crystals were detected in various bodily structures such as joints, cartilage, synovial bursa, tendons, or soft tissues (138). Gout and OA frequently coexist clinically in the same patient (139). However, the pathophysiologic relationship between the two remains elusive. A nested case-control study revealed that joints with a history of gout attacks were more likely to be affected by OA (140). Specifically, the distal interphalangeal joint of the finger, knee, mid-foot, and the first metatarsophalangeal joint showed a 12.7-fold, 3.1-fold, 2.9-fold, and 2.1-fold increased risk of clinically defined OA when attacked by gout, accompanied by chronic pain symptoms (141). A cross-sectional study, which included three cohorts of subjects aged 50 years or older with joint pain, found that individuals with gout had an increased risk of hand and foot OA but a decreased risk of knee OA (142). Another small cohort study focusing on middle-aged to elderly men with HUA, gout, or neither, revealed an elevated risk of knee OA in patients with gout, with no significant differences observed between patients with HUA and the other groups (143). Additionally, a cohort study involving 51,858 subjects and 1,435 cases of incident knee arthroplasty showed a significantly higher prevalence of knee arthroplasty among women with gout (24). Furthermore, a case-control study with 39,111 cases and 39,111 controls indicated that gout was associated with a 1-, 2-, 5-, and 10-year risk of OA (144).

Gout also promotes cartilage degradation due to the direct effects of MSU crystals. Studies have shown that joints (especially metatarsophalangeal joint, mid-foot, knee and finger distal interphalangeal joints) affected by acute attacks of gout also display clinical or radiographic features of OA (86, 87) suggesting that OA may predispose to the localized deposition of MSU crystals. It remains elusive whether gout promotes the development or progression of OA but aging and obesity remain as significant risk factors for both OA and gout. In a cross-sectional study, higher fat mass, increased muscle mass, and the presence of osteophytes were shown associated with the higher risk of tophaceous gout (145). Evidence from epidemiological studies supports an association between gout and OA (127). Compared to the painful flares of gout, the pain in OA is less intense and more variable. OA but not gout commonly affects hip, and wrist however OA and gout share some joints, like the distal interphalangeal joints, the first metatarsal phalangeal joints and knees (27). Poor renal excretion of urate is an important risk factor for gout but not for OA. OA genetics relates most commonly to cartilage biology and body mass index (BMI), whereas gout genetics relates largely to renal handling of urate. Men are more likely to develop gout, whereas women are more likely to develop OA indicating gender difference might play an important role in both gout and OA. UA has been hypothesized as a pathological link between gout and OA but current therapies for OA include only analgesia and joint replacement. The role of MSU crystal-induced inflammation in both OA and gout could be a possible shared pathway for pathogenesis. MSU and calcium pyrophosphate dihydrate (CPPD) crystal deposits in the talus were reported with cartilage degradation (146) suggesting these lesions are biomechanically induced. Soluble form of UA/urate in synovial fluid of knee OA was suggested to be strongly and positively associated with OA severity with pro-inflammatory cytokines like IL-1β and IL-18 (produced by uric acid-activated inflammasomes) in the synovial fluid (147). Thus, it was hypothesized that urate from systemic circulation or released from dying chondrocytes, might get into the joint and form micro-particles with proteoglycan released from dying cells. These micro-particles may then trigger the innate immunity and NALP3 inflammasome pathway (147). MSU crystals may trigger toll-like receptor 4 (TLR-4) primed by the reservoir of lipopolysaccharide (LPS) from gastrointestinal microbiome of obese people leading to phagocytosis, inflammasome activation, and subsequent inflammation and joint damage (148). Peripheral blood mononuclear cells (PBMCs), pre-treated with soluble UA and stimulated by MSU crystals, were shown to enhance IL-1β, IL-6 production (90) suggesting the condition of HUA may influence inflammatory responses by facilitating IL-1β production in peripheral blood leukocytes. IL-1β via activation of the MAPK (mitogen-activated protein kinase) can elevate the levels of matrix metalloproteinases that can cause cartilage matrix protein degradation (149). MSU is also linked to complement activation, direct T cell activation, and mast cell degranulation, all of which may be involved in the pathogenesis of OA (150, 151). The overlap between OA and gout/HUA suggests the efficacy of urate-lowering therapies for OA could be an interesting hypothetical strategy.

Previous research has established a reciprocal relationship between UA and OA (27). In a Korean longitudinal cohort study aimed at validating the potential link between gout and OA, it was found that over a 3-year period, SU levels in subjects without gout were inversely related to patellofemoral cartilage loss. However, SU was not identified as a risk factor for OA progression in the study, which was consistent with previous findings (136). Nonetheless, large-scale longitudinal studies involving diverse populations are needed to further elucidate this association (152). Lastly, an American cross-sectional study involving 2,213 individuals, including 412 with asymptomatic HUA, revealed that asymptomatic HUA was associated with an increased risk of OA, particularly in adults over the age of 60 without obesity (25) (**Table 3**). Clinical epidemiological studies on gout involving tendons are rare. Rotator cuff tears are a common cause of shoulder disability, and surgery is often chosen to restore function. In a 7-year Chinese longitudinal cohort study, 32,723 patients with gout and 65,446 control individuals participated at a 1:2 ratio. Patients with gout, especially those under 50 years old and not on hypouricemic medication, had a higher risk of needing rotator cuff repair surgery. Strict control of UA levels with hypouricemic medication could reduce this risk (153). Generally, SU levels should be kept below 6.0 mg/dL. For patients with tophi, initial SU levels should be below 5.0 mg/dL to dissolve tophi (138) (**Table 3**).

### 3.3 UA, spinal gout and IDD

#### 3.3.1 Spinal gout: a rare but serious disease involving MSU crystal deposit

Spinal gout, a rare disease, can often be mistaken for infections or neoplastic lesions, posing a challenge in diagnosis. In a case study, a patient with a long history of HUA and gout with progressive impairment of walking, MRI features revealed vascularized gouty tophi in the cervical spine in areas of low signal intensity both on T1 and T2 images and pathologic examination of the specimen demonstrated presence of fibrous tissue and amorphous MSU deposits and bone erosions by the MSU crystal deposits (154). In another case study, a 71-year-old man with a long history of HUA and gout (multiple tophi in elbows, hands, knees, and toes) with progressive quadriparesis (muscle weakness in both legs and both arms), severe cervical spondylosis and a herniated cervical disc at the C3-C4 level on MRI, was found to have intradiscal chalky white granular material called gouty tophus (155). Though spinal involvement in gout and cervical spinal cord compression caused by gout are uncommon, it should be considered in all patients presenting myelopathy and history of gout and HUA (156).

#### 3.3.2 UA: a potential risk factor for IDD

Intervertebral disc degeneration (IDD) is a common musculoskeletal disorder. Several case reports have described the presence of tophi in the endplate (a bilayer of cartilage and porous bone) or intervertebral disc (IVD) of patients with HUA or gout, and severe IDD. UA with its potent antioxidant activity helps maintain the stability of the IVD, but higher levels of UA production associated with generation of higher level of ROS (H_2_O_2_) production can lead to accelerate IDD (5). Moreover, MSU crystals accumulated in the endplate and IVD, can cause mechanical damage and inflammatory reactions that worsen IDD. In a recent study involving 1375 Chinese patients, it was found that there was a U-shaped relationship between SU levels and IDD, especially among men (26). The study also revealed a positive correlation between SU and lumbar spine BMD in American men within a certain range (SU < 5 mg/dL), although this correlation was no longer significant when SU levels exceeded 5 mg/dL. Factors such as race and age were found to influence this relationship (157). However, in a recent Mendelian randomization study, no causal relationship between SU and IDD was reported (158) (**Table 3**).

### 3.4 UA and bone remodeling

Bone remodeling takes place throughout life and is required not only for skeletal growth but also to maintain normal bone structure. Bone remodeling requires a fine balance between bone resorption by OC (osteoclast, differentiated from hematopoietic mononuclear cells) and bone formation by OB (osteoblast, differentiated from mesenchymal stem cells). Disruption of this balance leads to disease conditions like skeletal abnormalities, OP (osteophyte, bony lumps that grow on the bones in the spine or around joints) and rheumatoid arthritis (RA). Disorders in purine metabolism can lead to HUA with accumulation of ROS (H_2_O_2_) during UA biosynthesis. The pathological states induced by MSU and ROS, characterized by oxidative stress and inflammation, disrupt the balance of bone resorption and bone formation by inhibiting the expression of osteogenic factors, promoting OC differentiation, inducing bone erosion resulting to the loss of bone mass and destruction of bone microarchitecture (159). Previous research has shown a positive independent association between SU levels and BMD (bone mineral density), with a non-linear relationship observed in individuals with normal or low body weight. SU levels below 296 μmol/l may have a protective effect on BMD in normal- and low-weight OP patients, while levels above this threshold show no significant relationship with BMD (160). One study discovered a negative correlation between SU levels and total trabecular bone score (TBS) in adults (161). Maintaining SU at an optimal level can potentially help prevent OP and fractures. Interestingly, the association between SU levels and BMD at various skeletal sites differed in middle-aged and elderly men, with a positive relationship observed only in the normal weight group (162).

In Chinese patients with Parkinson’s disease (PD), higher SU levels within the normal range may serve as a biomarker for higher BMD and are associated with a lower prevalence of OP (163). Among hypertensive males, the relationship between SU and total femur BMD followed an inverted U-shaped curve, while in females, the association was predominantly negative. In the non-hypertensive group, the relationship between SU and total femur BMD also exhibited an inverted U-shaped curve across genders (164). Lumbar BMD showed a negative correlation with HUA in obese individuals, but this trend was observed only in men, not women. Furthermore, there was no significant link between hip BMD and HUA in obesity (165). SU levels were positively correlated with BMD at three different sites and were found to have a protective effect against abnormal BMD after adjusting for various confounders, including fat-free mass (FFM) and skeletal muscle mass index (SMI), in men aged 50 years and above (166). Among Mexican females, higher SU levels were associated with lower BMD at various skeletal sites (167). Additionally, individuals with HUA, gout, or varying levels of SU may have a higher risk of poor outcomes following hip fractures (168). In patients with T2D, higher SU levels were suggested to be a protective factor for bone health. However, the osteoprotective effects of SU were influenced by age and gender, being significant only in non-elderly men and elderly women (169). The SU-to-creatinine (UA/Cr) ratio was identified as an independent factor influencing bone turnover markers in patients with T2D (170).

## 4 Limitations and future perspectives

All the above studies unequivocally indicated that UA was clinically involved in musculoskeletal diseases, such as sarcopenia, gout, OA, RA, IDD, OP, EIAKI and others. The relationship between UA and many of musculoskeletal diseases still remained as part of a clinical discovery, analysis and summary. In this study, only clinical associations and some underlying mechanisms were mainly focused on and reviewed for indicating the potential links between UA and many musculoskeletal diseases. Moving forward, emerging evidences from epidemiological studies and newly discovered pathogenic pathways will contribute to a deeper understanding of the relationship between UA and musculoskeletal diseases. More and more animal studies and molecular biology experiments were expected to explain action mechanisms of UA influencing musculoskeletal functions. And more drugs and biotechnologies were urged to be developed for treating such musculoskeletal diseases associated with UA. Recently, an intestine-targeted explosive hydrogel microsphere has been developed to promotes UA excretion for gout treatment via an *in situ* small intestine mucosal layer regulation by oral microspheres (171). It may provide a brand-new pathway for the prevention and treatment of gout and other musculoskeletal diseases.

## 5 Conclusion

Nowadays, UA has been recognized as a significant risk factor for metabolic syndromes. Moreover, an increasing number of studies have highlighted the connection between UA and musculoskeletal diseases such as sarcopenia, OA, IDD, OP and EIAKI due to their prevalence (**Table 4**). This review presents the most recent and valuable insights into the mechanisms and associations between UA and musculoskeletal diseases, aiming to improve prevention and treatment strategies. In the context of preventing sarcopenia, both age and UA levels should be considered in adults, while gender and SU levels may directly impact muscle mass in children and adolescents. In the case of OA, higher SU levels and gout may serve as risk factors for OA progression, although there are conflicting reports on their relevance. As for IDD, there appears to be a U-shaped relationship between UA and IDD, particularly among Chinese men. And maintaining SU levels within a certain range may help prevent OP and fractures (**Table 4**). Various mechanisms, such as oxidative stress, deposition of MSU crystals, and inflammation, have been proposed to explain the effects of HUA in these conditions. Emerging evidences from epidemiological studies, together with recently discovered pathogenic pathways, drugs and biotechnologies will help to solve the therapeutic puzzle troubling UA related musculoskeletal diseases.

**Table 4:** Summary of key information on clinical associates between UA and musculoskeletal diseases.

Main disease	UA levels	Features	Ref.
Sarcopenia	High	abnormally high SU levels, sarcopenia	(91)
T2D and sarcopenia	High	higher SU, an independent risk factor of reduced muscle mass in men with T2D	(93)
MCD	High	blocked glycogenolysis, myogenic HUA and increased oxidative stress via the xanthine oxidase pathway	(92)
EIAKI	Low	one of RHUC complications, loin pain, nausea and vomiting after exhaustive (anaerobic) exercise	(32)
Rotator cuff tears	High	patients with gout, a relatively higher risk of receiving rotator cuff repair surgery	(153)
OA	High	gout, involved in knee pain, joint space narrowing and total knee replacement OA; OA in gout patients, more serious	(24, 27, 86, 133, 134, 141, 143, 144)
IDD	Low and High	both lower and higher SU levels, the LDH rate higher, a U-shaped relationship between SU and IDD, especially among men	(26)
OP	High	maintaining SU at an optimal level can potentially help prevent OP and fractures	(160–162)

HUA, hyperuricemia; IDD, intervertebral disc degeneration; LDH, lumbar disc herniation; OA, osteoarthritis; OP, osteoporosis; RHUC, renal hypouricemia; SU, serum urate; UA, uric acid.
